# IL-10 Dysregulation in Acute Mountain Sickness Revealed by Transcriptome Analysis

**DOI:** 10.3389/fimmu.2017.00628

**Published:** 2017-05-30

**Authors:** Bao Liu, Jian Chen, Long Zhang, Yixing Gao, Jianhua Cui, Erlong Zhang, Gang Xu, Yan Liang, Yu Liang, Jian Wang, Yuqi Gao

**Affiliations:** ^1^Institute of Medicine and Hygienic Equipment for High Altitude Region, College of High Altitude Military Medicine, Third Military Medical University, Chongqing, China; ^2^Key Laboratory of High Altitude Medicine, PLA, Chongqing, China; ^3^Key Laboratory of High Altitude Environmental Medicine, Third Military Medical University, Ministry of Education, Chongqing, China; ^4^BGI-Shenzhen, Shenzhen, China; ^5^Research Center of PLA for Prevention and Treatment of High Mountain Sickness, The 18th Hospital of PLA, Xinjiang, China

**Keywords:** interleukin-10, acute mountain sickness, RNA-seq, inflammatory response, immune response

## Abstract

Acute mountain sickness (AMS), which may progress to life-threatening high-altitude cerebral edema, is a major threat to millions of people who live in or travel to high altitude. Although studies have revealed the risk factors and pathophysiology theories of AMS, the molecular mechanisms of it do not comprehensively illustrate. Here, we used a system-level methodology, RNA sequencing, to explore the molecular mechanisms of AMS at genome-wide level in 10 individuals. After exposure to high altitude, a total of 1,164 and 1,322 differentially expressed transcripts were identified in AMS and non-AMS groups, respectively. Among them, only 328 common transcripts presented between the two groups. Immune and inflammatory responses were overrepresented in participants with AMS, but not in non-AMS individuals. Anti-inflammatory cytokine IL10 and inflammation cytokines IF17F and CCL8 exhibited significantly different genetic connectivity in AMS compared to that of non-AMS individuals based on network analysis. IL10 was downregulated and both IF17F and CCL8 were upregulated in AMS individuals. Moreover, the serum concentration of IL10 significantly decreased in AMS patients after exposure to high altitude (*p* = 0.001) in another population (*n* = 22). There was a large negative correlation between the changes in IL10 concentration, *r*(22) = −0.52, *p* = 0.013, and Lake Louise Score. Taken together, our analysis provides unprecedented characterization of AMS transcriptome and identifies that genes involved in immune and inflammatory responses were disturbed in AMS individuals by high-altitude exposure. The reduction of IL10 after exposure to high altitude was associated with AMS.

## Introduction

Acute mountain sickness (AMS) is the most common disease caused by the lower pressure and reduced oxygen amounts at high altitudes (above 2,500 m) and can progress to high-altitude cerebral edema in severe cases, which has a high mortality (1, 2). A significant increase in the number of sojourns and mountaineers has been observed in high altitudes (3, 4). Although the awareness of altitude-related health hazards increased, the prevalence of AMS was ~16–100% in different study designs (3, 5, 6); the median of AMS incidences without prophylaxis was 60% in randomized trails (7).

Hypoxia is a principal etiological factor for AMS and initiates a pathophysiology process and causes its symptoms. Insufficient cerebrospinal compliance (8), alterations in fluid balance (9), activation of nociceptors induced by free radicals (10), and vasogenic edema caused by increased capillary permeability (11, 12) have all been associated with AMS development, but the biological pathways and exact molecular mechanisms underlying AMS remain unknown (13).

Comparison of transcriptome differences in samples from individuals with and without AMS can reveal distinct differences in gene regulation changes related to AMS at genome-wide level. Transcriptome of whole blood is supposed to constitute an accessible window to the multiorgan transcriptome by several transcriptome profiling studies of different conditions and diseases (14, 15). In this study, we used RNA sequencing to create whole blood transcriptomes to assess the gene expression landscape in response to acute altitude hypoxia and used this to define the molecular changes involved in AMS.

## Materials and Methods

### Study Oversight

The study was reviewed and approved by the Third Military Medical University Ethics Committee, China. Excluding people with lung, heart, and blood diseases, 10 individuals were recruited to provide samples for RNA sequencing, another population encompassed 22 individuals were constituted for protein validation set. All of them were healthy young men who had never previously been at high altitude. The study was thoroughly explained to all individuals who agreed to participate, and all participants signed informed consent forms before their examinations.

### Study Procedure

Volunteers for transcriptomic analyses set were assembled at a low-altitude starting point (1,300 m), then made a rapid ascent to a high altitude (5,300 m) by bus over a 72-h period, including a 1-day stopover at 3,000 m. Individuals for protein validation set traveled by train from 300 to 3,658 m in ~48 h. Vital signs, including heart rate, blood pressure, and oxygen saturation, were measured at 7:00 p.m. prior to ascent. Upon arrival at high altitudes, the same measurable parameters were performed at 7:00 p.m. daily for 5 days, and mean values were calculated. During the exposure to high altitudes, all volunteers did the regimented daily life and avoided any exercises or physical labor.

Blood was drawn from a peripheral vein in the morning soon after waking before ascending to high altitudes and after 3 days high-altitude exposures. All blood samples of RNA sequencing set were stored at −80°C with RNA protecting tube for subsequent analysis. Serum samples of validation set were separated at 3,000 rpm and stored at −80°C for subsequent use.

**Figure 1:**
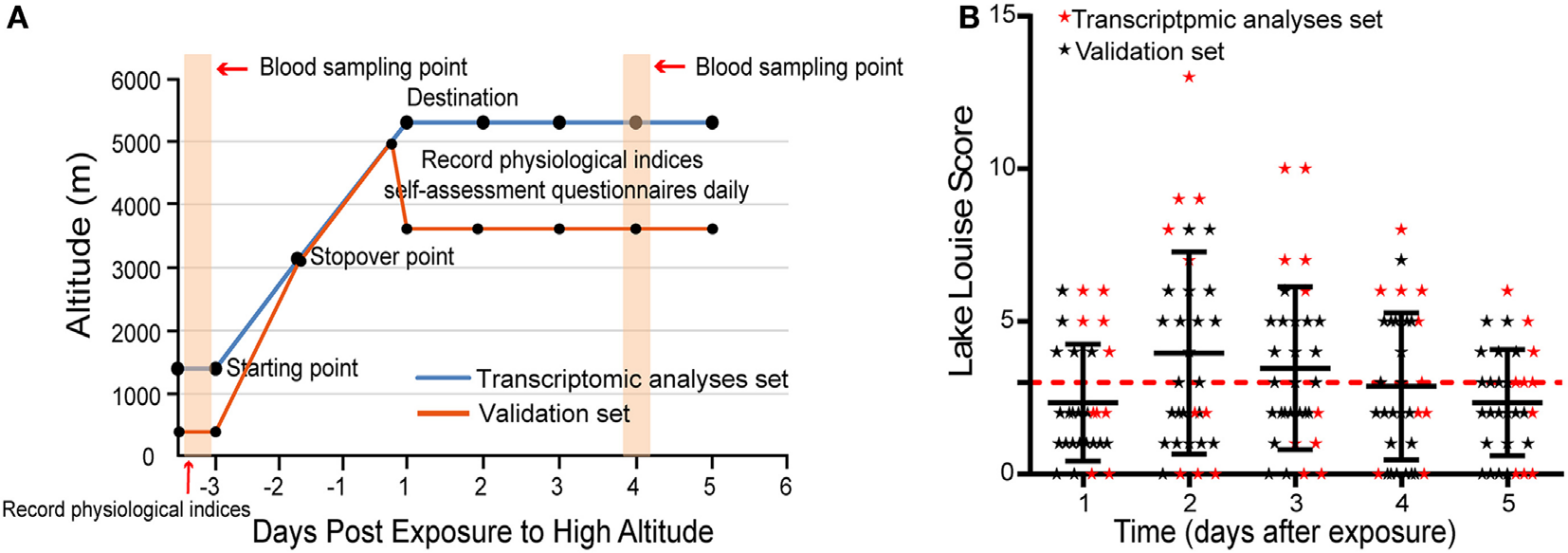
**The distribution of Lake Louise Scores and symptoms during the investigation period**. **(A)** Schematic of study procedure. **(B)** Scatter plot for the LLS of all individuals. The numbers correspond to the days when acute mountain sickness (AMS) was assessed. The red dashed line denotes the threshold of diagnosis for AMS.

Self-assessment questionnaires of the Lake Louise Scoring System (16) were performed at 8:00 a.m. daily for 5 days when volunteers had arrived at high altitudes. AMS was diagnosed through the use of Lake Louise Scoring System, which comprises a questionnaire and a scorecard that determine severity. Individuals with a Lake Louise Score (LLS) ≥ 3 (including a headache score of ≥1) were designated as having AMS, while individuals without a headache or with LLS < 3 were considered to be non-AMS. More clear study procedure is depicted in Figure 1A.

### RNA Sequencing and Analysis

Total RNAs were extracted from 20 whole blood samples from individuals before and after exposure to high altitude. mRNA-seq libraries were constructed according to the TruSeq RNA Sample Prep Kit v2 (Illumina) and sequenced on an Illumina HiSeq 2000 sequencer, following the manufacturer’s instructions. We used the following criteria to filter the raw reads sequenced from the Illumina HiSeq 2000 sequencer: (1) remove reads with adaptors; (2) remove reads in which unknown bases are more than 10%; and (3) remove low-quality reads (the percentage of low-quality bases is over 50% in a read, we define the low-quality base to be the base whose sequencing quality is not more than 5). Then, clean reads were aligned to human genome build hg19 using TopHat (v1.4.1) (17), which built on the ultrafast short read mapping program Bowtie (v1.1.2) (18). The basic information of RNA-seq data set is listed in Table S1 in Supplementary Material.

We used reads per kilobase million as a normalization method in following analysis (19). The expression value of each transcript was calculated; differentially expressed transcripts were selected by comparison of postexposure and preexposure expression values in each group using a density-based pruning algorithm whose key idea is based on the observation that differentially expressed genes tend to have average expression values across conditions. And it confirmed that differentially expressed genes between two conditions are usually located in the boundary region in the 2D feature space of average gene expression versus average difference of gene expression (20).

We used the gene ontology (GO) tool DAVID (version 6.8 Beta) (21) to determine enriched GO terms in the differentially expressed gene set. Then, the Gene Functional Classification tool from the DAVID suite was used to cluster enriched GO terms (biological processes) with the classification stringency set to medium and all other options set to default. According to the instructions of DAVID, we selected the groups with a higher score (scores ≥ 1.3) for subsequent analysis, which indicates that the gene members in the group are involved in more important (enriched) terms in a given study.

To further investigate the potential functional interactivity between gene products and compare the different regulatory modes between AMS and non-AMS groups, we used the biological modules condensed from enriched GO terms to construct gene networks for each group within differentially expressed genes of participants with AMS.

We investigated five biological modules including immune response, inflammatory response, leukocyte activation, sprouting angiogenesis, and response to oxidative stress, which associated with the inflammatory and immune responses under hypoxia (22–24). For both AMS and non-AMS groups, expression data from high altitude of genes in these modules were used to calculate the topological overlap matrix from given expression data to get the topological overlap between xi and xj to measure clustering or shared neighbors (25). The connections among genes were depicted using VisANT (26). The connectivity, which is defined as the sum of a gene’s connection strengths with all other genes in the network, was compared between AMS and non-AMS groups of all genes within their respective network.

### Enzyme-Linked Immune Sorbent Assay (ELISA)

We measure the concentration of IL10, IL17F, and CCL8 in plasma collected from individuals in transcriptomic analyses set using ELISA with commercially available kits (product number: IL10, ELH-IL10-1, IL17F, ELH-IL17F-1, CCL8, ELH-MCP2-1, RayBiotech, GA, USA) according to the manufacturer’s instructions.

### Measurement of Serum IL10 with Clinical Diagnosis Assay

The protein expression of IL10, which exhibited significantly different genetic connectivity between AMS and non-AMS groups, was analyzed before and after arrival at high altitude with chemiluminescent immunometric assay using an IMMULITE^®^ 1000 autoanalyzer (Siemens, Germany) with commercially available kits (catalog number: LKXPZ, Siemens Healthcare Diagnostics Products Ltd.), which is a method of diagnostic use in the study of inflammatory disease.

### Statistical Analysis

Statistical analyses were performed with the use of SPSS software, version 19·0. Normality was assessed for all data sets by the Shapiro–Wilk’s test. To examine the difference between the physiological indices of AMS and non-AMS groups, a one-way ANOVA with repeated measures was employed. Age and AMS severity were calculated with an independent *t*-test. Paired *t*-test or Wilcoxon signed-rank test analysis was used to compare IL10, IL17F, and CCL8 protein expression of AMS and non-AMS individuals before and after exposure to high altitude, respectively. A Pearson’s product–moment correlation was run to assess the relationship between the changes in IL10 concentration and LLS score in individuals of protein validation set.

## Results

### Clinical Characteristics of the Individuals

In transcriptomic analysis set, five individuals were diagnosed as AMS and five as non-AMS, while 12 volunteers were designated as AMS and 10 as non-AMS in validation set (Table S2 in Supplementary Material). The onset of symptoms commenced 3–24 h after arrival at the highest altitude, peaked in severity between 48 and 72 h (Figure 1B). During the 5 days of the study, headaches were the most common symptom.

We found that rapid ascent to a high-altitude environment initiated a cascade of physiological responses: oxygen saturation decreased substantially once individuals were at high altitude; heart rate and blood pressure increased; the mean hemoglobin concentration was higher in individuals postexposure to high altitude in contrast to preexposure to high altitude. But, changes in these clinical features have no significant difference between AMS and non-AMS groups except AMS severity (Table 1).

**Table 1:** **Demographic data and physiological indices of study subjects and comparison in AMS and non-AMS groups**.

Transcriptomic analyses set				Protein validation set			
Variable	Non-AMS group (*N* = 5)	AMS group (*N* = 5)	*P*-value	Non-AMS group (*N* = 10)	AMS group (*N* = 12)	*P*-value
Age, years			0.35			0.31
Median	22	21		24	23.5	
Range	20–23	20–23		22–32	21–26	
SpO_2_ (%)			0.12			0.63
Plain	97 (1.0)	96 (2.2)		98 (0.8)	98 (0.5)	
Plateau	81 (1.2)	77 (7.4)		89 (1.5)	89 (1.8)	
HR (beats/min)			0.06			0.40
Plain	65 (10.1)	76 (13.8)		69 (5.6)	64 (10.8)	
Plateau	89 (7.3)	101 (14.8)		87 (9.0)	86 (9.7)	
SBP (mmHg)			0.96			0.74
Plain	116 (15.0)	116 (14.4)		113 (8.3)	111 (8.9)	
Plateau	124 (12.2)	125 (13.4)		127 (5.9)	128 (8.7)	
DBP (mmHg)			0.60			0.91
Plain	58 (5.7)	60 (5.5)		66 (5.6)	69 (6.9)	
Plateau	74 (7.6)	75 (7.4)		77 (5.4)	76 (6.6)	
Hemoglobin (g/L)			0.92			0.14
Plain	141 (14.7)	140 (5.1)		153 (8.4)	149 (6.9)	
Plateau	158 (11.5)	160 (6.2)		168 (7.7)	163 (10.8)	
AMS severity						
LLS	2.0 (1.2)	9.4 (2.3)	<0.001	1.9 (0.6)	5.8 (1.1)	<0.001

*Values are mean (SD). A *P*-value of less than 0.05 was considered to indicate statistical significance. *P*-Values were calculated with the use of one-way ANOVA with repeated measures, except for age and AMS severity, which were calculated with the unpaired *t*-test*.

*SpO_2_, blood oxygen saturation; AMS, acute mountain sickness; HR, heart rate; SBP, systolic blood pressure; DBP, diastolic blood pressure; LLS, Lake Louise Score*.

### Identification of Distinct Genetic Expression Patterns in the AMS Group

**Figure 2:**
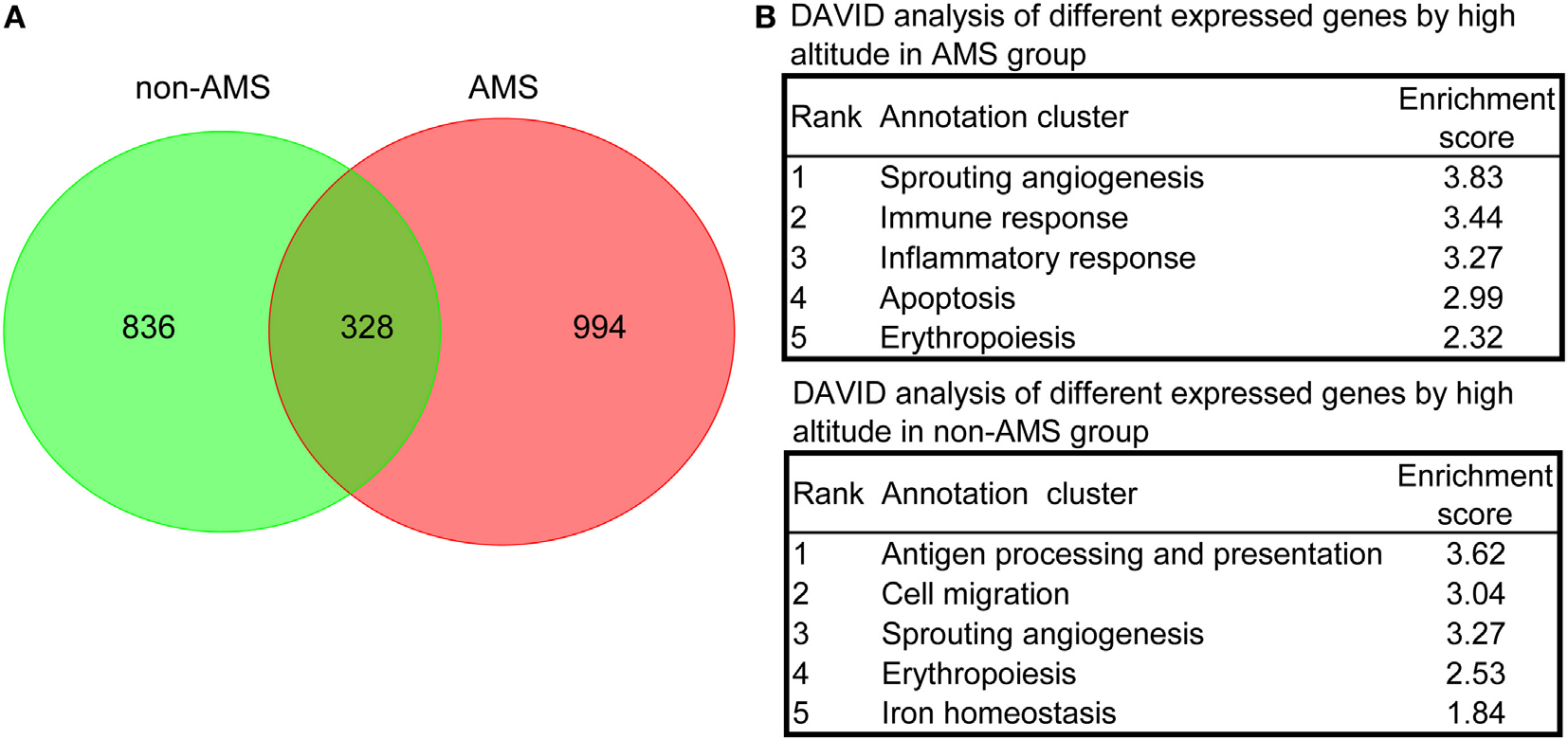
**Differentially expressed transcripts and overrepresented biological processes in non-acute mountain sickness (AMS) and AMS groups**. **(A)** Venn diagram showing the number of transcripts equally and differentially expressed in whole blood cells derived from non-AMS and AMS individuals. **(B)** The top five representative biological modules of differentially expressed transcripts for non-AMS and AMS groups.

We carried out further analyses on those transcripts that showed expression pattern differences in the AMS and non-AMS transcriptome landscapes to uncover the pathophysiological process of AMS. The results of a density-based pruning algorithm analysis of the transcriptome data for each group pre- and postexposure to high altitude showed that 1,164 and 1,322 transcripts differentially expressed in the non-AMS and AMS groups, respectively (Tables S3 and S4 in Supplementary Material). Among them, only 328 common transcripts presented between non-AMS and AMS groups (Figure 2A), indicating a substantially different signature of gene expression regulation between the two groups.

We used functional annotation clustering analysis to determine the biological module for the differentially expressed transcripts and found that the genes whose transcripts were differentially expressed in the AMS group pre- and postexposure to high altitude were more enriched in the biological processes relating to sprouting angiogenesis, immune responses, inflammatory responses, apoptosis, and erythropoiesis. In contrast, genes whose transcripts were differentially expressed in the non-AMS group were associated with antigen processing and presentation, cell migration, sprouting angiogenesis, erythropoiesis, and iron homeostasis (Figure 2B; Table S5 in Supplementary Material). Intriguingly, there were no genes enriched in inflammatory and immune responses in the non-AMS group. Moreover, pre- and postexposure to high altitude, a change in the interleukin genes occurred in individuals with AMS, with downregulation of *IL2, IL4, IL6ST, IL7, IL7R, IL10, IL17B, IL32*, and *IL23R* and upregulation of *IL13* and *IL17F*. The results indicated that inflammatory and immune responses are important pathophysiological processes in AMS.

**Figure 3:**
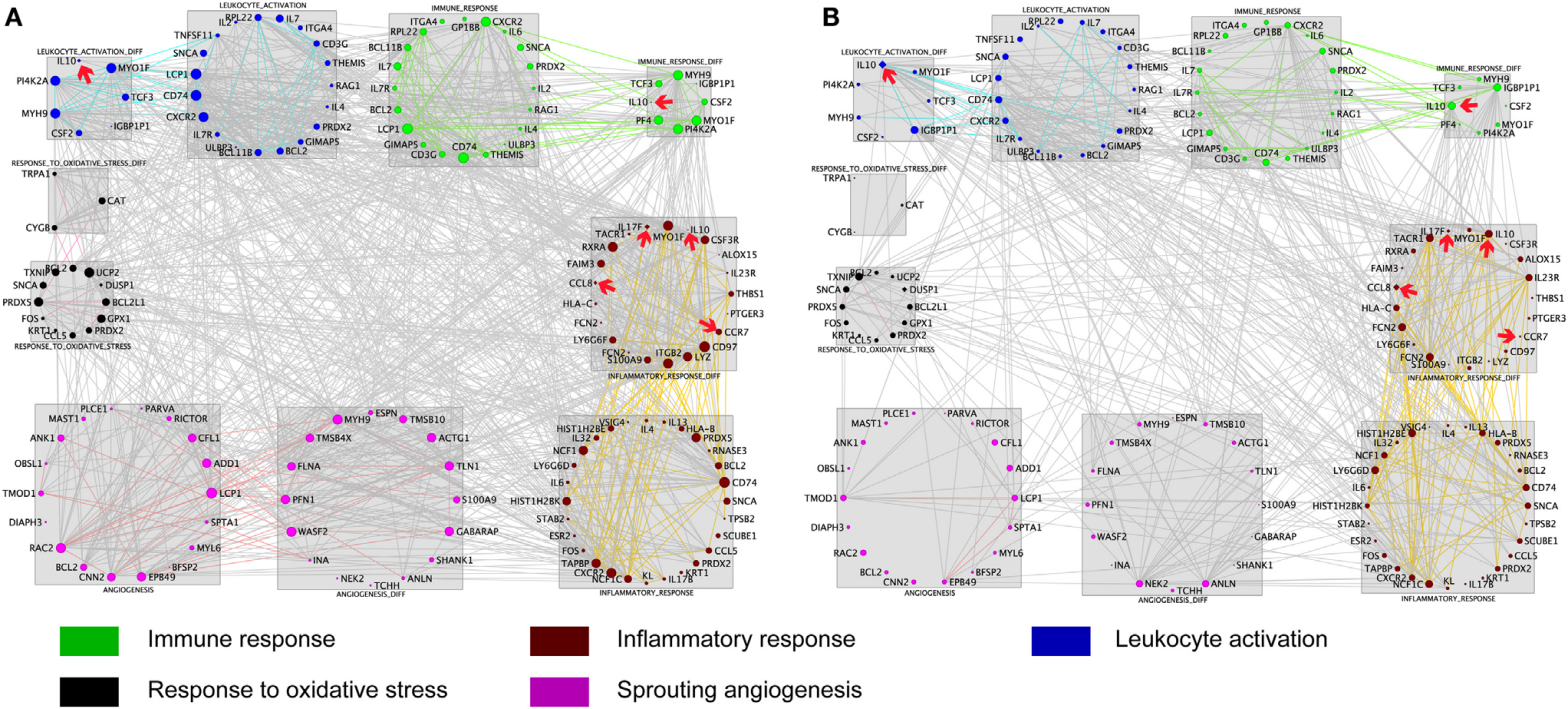
**Cluster visualization identifies acute mountain sickness (AMS)-specific transcriptional regulatory patterns**. **(A)** Connections of differentially expressed genes enriched in five functional clusters that were overrepresented in AMS are depicted. **(B)** Connections from A that are present in the AMS group but absent in the non-AMS group. Node color represents the enriched biological process. Node size reflects the number of direct connections a gene has within the network. Genes enriched in different clusters are connected by gray lines, and genes enriched in each cluster are connected by colored lines. The cytokines with significant differential connectivity are indicated by red arrows.

To further explore the AMS-specific transcriptional regulatory patterns for inflammatory and immune responses, we constructed networks in AMS and non-AMS groups. We further calculated the connectivity value for each gene in the networks on the basis of the AMS and non-AMS expression data from high altitude. In the AMS group, the strongest connections were present among five functional annotation clusters in the network (Figure 3A). To determine whether these connections were also present in the non-AMS group network, we looked at the network conservation. The connections that showed gene coexpression relationships in the AMS group were essentially absent in the non-AMS group (Figure 3B).

**Figure 4:**
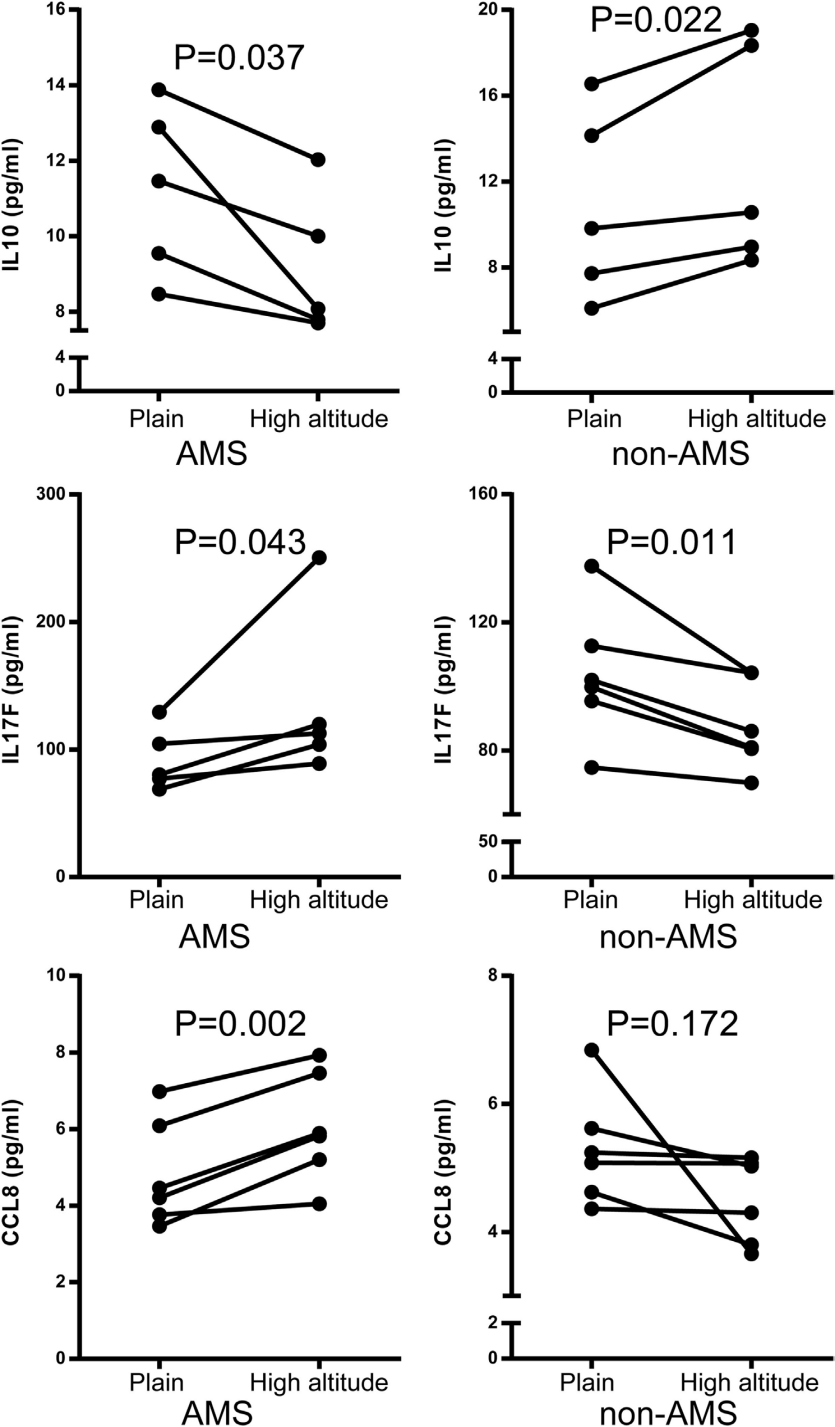
**Quantification of IL10, IL17F, and CCL8 protein using enzyme-linked immunosorbent assay acute mountain sickness (AMS) (*N* = 5) and non-AMS (*N* = 5) individuals in transcriptome assay set before and after exposure to high altitude, respectively**.

Differential connectivity reflects a distinct pattern of genetic expression. We examined the connectivity values of the genes in networks of the AMS and non-AMS groups and made a comparison. We identified 43 genes with significant differences between the two groups (Table S6 in Supplementary Material). Specifically, the cytokines *IL10, CCL8, CCR7*, and *IL17F* possessed substantial differential connectivity between AMS and non-AMS groups (Figure 3). In the AMS group, *CCL8* and *IL17F* present an upregulation and downregulation of IL10 and *CCR7* (Figure 4; Table S4 in Supplementary Material). And we found that *dual-specificity protein phosphatase* (*DUSP1*), involved in the production of IL10 ^26^, has an upregulation in the AMS group, but its homolog *DUSP19* is downregulated in the non-AMS group. Taken together, our results show that individuals with AMS have a distinct genetic profile in which anti-inflammatory cytokine production was reduced.

### Serum IL10 in AMS and Non-AMS Groups

A meta-analysis in our laboratory has demonstrated that dexamethasone, an immunosuppressive drug, has been shown to be quite effective in preventing and treating AMS (27). Moreover, cell assays demonstrated that dexamethasone can promote the production of IL10 (28, 29). Combined with our transcriptome analyses results, we speculated that IL10 production may be associated with AMS.

**Figure 5:**
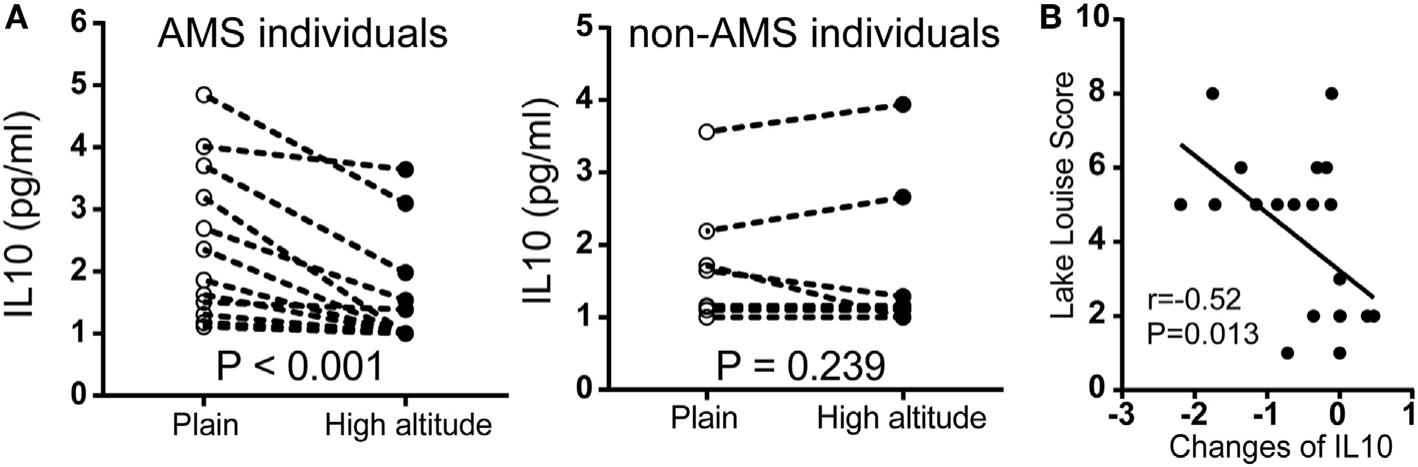
**Comparison of IL10 protein expression in independent non-acute mountain sickness (AMS) and AMS individuals by paired *t*-test**. **(A)** Illustration of IL10 expression in individuals with AMS (*N* = 12) and non-AMS (*N* = 10), respectively, before and after exposure to elevated altitudes. **(B)** Pearson correlation for the changes in IL10 concentration (IL10 concentration postexposure to high altitude − IL10 concentration preexposure to high altitude) and Lake Louise Score.

To further assess our findings with cytokine expression, we measured serum IL10 by chemiluminescent immunometric assay in another population encompassed 22 individuals. In participants with AMS, IL10 protein expression was significantly downregulated after exposure to high altitude. By contrast, there were no significant changes in non-AMS individuals (Figure 5A). There was a large negative correlation between the changes in IL10 concentration (IL10 concentration postexposure to high altitude − IL10 concentration preexposure to high altitude), *r*(22) = −0.52, *p* = 0.013, and Lake Louise Score (Figure 5B).

## Discussion

In the present study, we performed RNA sequencing in whole blood cells of individuals who were rapidly exposed to high altitudes and analyzed the sequencing data of individuals before and after high-altitude exposure in AMS and non-AMS groups. Inflammatory response and immune response were specific transcriptional alteration that occurred in participants with AMS, as revealed by functional annotation analyses of the differentially expressed transcripts.

It has been demonstrated in previous studies that inflammatory response is associated with AMS (30, 31). In this study, we have illustrated the questions using a system-level methodology and demonstrated that genes associated with immune and inflammatory response, including chemokine and their receptors, CD and HLA molecules, and inflammatory cytokines were disturbed in AMS individuals by high-altitude exposure acutely. Among them, the anti-inflammatory cytokine IL10 presents significantly different connectivity between AMS and non-AMS individuals and the changes in IL10 after exposure to high altitude present strong correlation with AMS. In our recent study, we found that the concentration of inflammatory cytokines presents positive correlation with AMS, such as IL6, TNF-α, and IL-1β (32). Maybe the increase in these inflammatory cytokines was a result of the decrease in production of anti-inflammatory cytokine IL10.

The molecular signals for IL10 production were observed in the transcriptional landscape. *DUSP1* is enriched in response to oxidative stress and limits IL10 production by negatively regulating p38 phosphorylation (33). It has been shown to be induced by hypoxia and has antioxidant properties (34). We note here that *DUSP1* is upregulated in our AMS group, but *DUSP19*, a homolog of *DUSP1*, was downregulated in the non-AMS group. Another IL10 regulatory mechanism involves the inhibition of T-cell differentiation, which normally secretes IL10. One protein that is involved in the maintenance of Treg cell function is CCR7 (35), and this was downregulated in the AMS group. In contrast, its ligand CCL19 was upregulated in the non-AMS group. These findings suggested that the IL10 production could be diminished in participants with AMS both through the signaling molecule DUSP1 and by genes that control T cell differentiation.

Immune system is a highly regulated system that is sensitive to several extrinsic factors including environmental stress (36). Consistently, previous data from human studies and animal models indicate that T-cell function is dampened following exposure to hypoxia (37, 38). The concept that hypoxia can induce inflammation has also gained credence in recent studies (39). Especially, hypoxia coupled with reduced intrinsic IL10 could activate angiogenesis pathway excessively and result in disease (40, 41). The present study observed that hypoxia could be as a driver for immune and inflammatory responses in AMS patients and revealed that the reduction of anti-inflammatory cytokine IL10, after high altitude exposure, was associated with AMS.

IL10 is a central cytokine during the resolution phase of inflammation and a general suppressor of cytokines that inhibit pro-inflammatory responses from the innate and adaptive immune pathways and prevents tissue lesions caused by exacerbated adaptive immune responses (42). Suppression of the anti-inflammatory response in the AMS group was accompanied by a decrease in production of IL10. In the absence of countervailing regulation, the inflammatory signal induced by hypobaric hypoxia is converted and readily amplified in an organism, and leukocytes undergo continuous cellular rolling and adherence to endothelium promoted by chemokine, such as CCL8 (43) upregulated in AMS. This results in increased vascular permeability and ultimately leads to clinical manifestation consequent to vasogenic edema (44, 45).

Dexamethasone, an immunosuppressive drug, has been shown to be quite effective in preventing and treating AMS (27), although the mechanism by which this is achieved is unclear. Recent studies were reported that dexamethasone can promote the production of IL10 (28, 29). Results from our study demonstrated that IL10 may be a key point for AMS, and thus may be relevant to the effectiveness of dexamethasone. However, dexamethasone is a rather non-specific, generalized suppressor of inflammation. Thus targeted therapy with IL-10 might be more beneficial in AMS prevention.

We demonstrated that inflammatory response and immune response were specific transcriptional alterations that occurred in participants with AMS and anti-inflammatory cytokine IL10 reduction presents a large positive correlation with AMS; the more the reduction of IL10, the more the severity of AMS. However, only the young health men were included in this study because they are the main part of population who travel to high altitudes for recreation, work, and pilgrimage. Therefore, further investigations in more individuals to confirm these results to exclude potential bias should include females, high altitude residents and old age people. And the more detailed mechanism of IL10 reduction in AMS after exposure to high altitude need to be studied in future.

## Conclusion

In summary, we have explored the gene expression pattern of AMS pattern at currently the most detailed level of resolution. Our data suggest that inflammatory response and immune response were specific transcriptional alterations that occurred in participants with AMS. Cytokine regulatory changes are associated with AMS, and anti-inflammatory cytokine IL10 reduction presents a large positive correlation with AMS; the more the reduction of IL10, the more the severity of AMS. Thus, targeted therapy with IL-10 might be more beneficial in AMS prevention.

## Data Accession

The raw data have been deposited to Gene Expression Omnibus (GEO) under accession GSE75665.

## Ethics Statement

The study was reviewed and approved by the Third Military Medical University Ethics Committee, China. Excluding people with lung, heart, and blood diseases, we recruited, enrolled, and obtained informed consent from 32 healthy young male volunteers, who had never previously been at high altitude.

## Author Contributions

YG, YL, and JW conceived and designed the study. YG and YL oversaw laboratory analyses and JC provided the overall supervision of the study. LZ, BL, YG, YL, and EZ did the laboratory experiments or contributed to the statistical analysis, or both GX and JC contributed to sample and physical data collections. JC, BL, and LZ drafted the report. All the authors contributed to the interpretation of results, critical revision of the manuscript, and approved the final manuscript. YG is the guarantor.

## Conflict of Interest Statement

The authors declare that the research was conducted in the absence of any commercial or financial relationships that could be construed as a potential conflict of interest.
